# Nanoparticle encapsulation of non-genotoxic p53 activator Inauhzin-C for improved therapeutic efficacy

**DOI:** 10.7150/thno.57404

**Published:** 2021-05-12

**Authors:** Nimisha Bhattarai, Jieqiong Wang, Daniel Nguyen, Xiaoxiao Yang, Linh Helmers, Jennifer Paruch, Li Li, Yiwei Zhang, Kun Meng, Alun Wang, Janarthanan Jayawickramarajah, Binghe Wang, Shelya Zeng, Hua Lu

**Affiliations:** 1 Department of Biochemistry & Molecular Biology and Cancer Center, Tulane University School of Medicine, New Orleans, LA, USA.; 2 Department of Chemistry, Georgia State University, Atlanta, GA, USA.; 3 Laboratory of Cellular Immunology, Ochsner Clinic Foundation, New Orleans, LA 70121, USA.; 4 Department of Chemistry, Tulane University School of Science and Engineering, New Orleans, LA, USA.; 5 Department of Pathology, Tulane University School of Medicine, New Orleans, LA, USA.

**Keywords:** p53, nanoparticle encapsulation, Inauhzin-C, PK, cell growth, efficacy, anti-cancer therapy, lung cancer, and colorectal cancer

## Abstract

The tumor suppressor protein p53 remains in a wild type but inactive form in ~50% of all human cancers. Thus, activating it becomes an attractive approach for targeted cancer therapies. In this regard, our lab has previously discovered a small molecule, Inauhzin (INZ), as a potent p53 activator with no genotoxicity.

**Method:** To improve its efficacy and bioavailability, here we employed nanoparticle encapsulation, making INZ-C, an analog of INZ, to nanoparticle-encapsulated INZ-C (n-INZ-C).

**Results:** This approach significantly improved p53 activation and inhibition of lung and colorectal cancer cell growth by n-INZ-C *in vitro* and *in vivo* while it displayed a minimal effect on normal human Wi38 and mouse MEF cells. The improved activity was further corroborated with the enhanced cellular uptake observed in cancer cells and minimal cellular uptake observed in normal cells. *In vivo* pharmacokinetic evaluation of these nanoparticles showed that the nanoparticle encapsulation prolongates the half-life of INZ-C from 2.5 h to 5 h in mice.

**Conclusions:** These results demonstrate that we have established a nanoparticle system that could enhance the bioavailability and efficacy of INZ-C as a potential anti-cancer therapeutic.

## Introduction

The p53 pathway has been a widely investigated target for anti-cancer therapy as it has a major effect on several cellular processes, such as cell cycle arrest, apoptosis, autophagy, senescence, cell migration, metabolism and angiogenesis 1-3. Several small molecules, such as Nutlin, Prima-1, and Tenovin 1 and 6, have been developed in an attempt to target this pathway in various ways 2, 4. These small molecules increase levels of p53 within the cancer cell through various mechanisms, ultimately resulting in cell death. For example, Nutlin blocks MDM2/p53 binding, preventing ubiquitylation of the latter and increasing its levels 5. Tennovin 1 and 6 inhibit SIRT 1 and SIRT 2 deacetylases preventing deacetylation of p53, and ultimately resulting in increased levels of p53 4, 6. A class of anthraquinones has been shown to induce MDM2 degradation as a way to upregulate p53 7, 8. While several of these small molecules have been developed, investigated and employed for anti-cancer activity, many of them are insoluble in water which limits many of their bio-applications 9.

Nanomedicine has widely been investigated for enhancing the drug bioavailability and for developing targeted therapies. These nanoparticles can be comprised of lipids, peptide/drug conjugates, polymers, and metal components. Lipid based nanocarriers have been employed for both mRNA based cancer therapy and the SARS-CoV-2 mRNA vaccine delivery. In both cases, the lipid nanoparticle is known to protect the nucleic acid from degradation 10, 11. Several peptide based nanoparticles have been developed for activation of p53 and have shown promising activity 12-15. Other studies demonstrate promising *in vitro* and *in vivo* applications of drug encapsulated within organic/polymer-based nanodrug delivery systems 9, 16-19. In this regard, chitosan is a polymer that has been widely used for nanodrug fabrication due to its biocompatibility, low toxicity, high stability and ability for versatile routes of administration 20-22. Cyclodextrin (CD) is a cyclic oligomer that has also been employed nanodrug delivery and has shown great promise in enhancing the bioavailability of lipophilic drugs through drug encapsulation. β-CD has been the most widely investigated for drug encapsulation due to its optimal cavity size, high encapsulation efficiency and low cost 20, 23-25. Addition of chemical modifications, such as hydroxypropyl (HP) and methyl groups, further aid in the solubility of CD, and thus these modified β-CD's (HP-β-CD and methylated β-CD) are widely used for lipophilic drug complexation to enhance the bioavailability 23, 24, 26. Several groups have also investigated the efficacy of nanodrugs fabricated from a combination of chitosan and cyclodextrin 27-30. Recently, a study has demonstrated that encapsulation of the cyclodextrin/paclitaxel host/guest complex into chitosan-based nanoparticles enhanced the *in vivo* half-life of paclitaxel by 1.5 fold 31. Furthermore, chitosan/cyclodextrin nanoparticles provide the distinct advantage of employing both CD drug encapsulation to enhance drug solubility and the stability and biocompatibility of chitosan based nanoparticles.

Our lab has recently developed a small molecule p53 activator INZ that can inhibit SIRT-1 deacetylase activity, preventing degradation of p53 through the p53/MDM2 negative feedback loop, and ultimately increasing the level of p53. Intriguingly, while INZ displayed significant activation of p53 in cancer cells, minimal to no p53 induction was observed in normal cells 4. Moreover, while most chemotherapeutics employ DNA damage as their mechanism of action, INZ was found to be a nongenotoxic p53 activator 32, 33. Investigation of INZ was furthered by developing several analogs of INZ and assessing their *in vitro* activity to determine the role of structure on activity of this molecule. One analog INZ-C had the most improved IC_50_ and p53 induction; thus, in the study presented here, we will focus on this analog 34. As shown in our previous studies, INZ-C had activity towards p53 wt and null cell lines with minimal to no activity in wi38 normal fibroblast cell lines 34. However, despite its promising *in-vitro* activity, similar to other lipophilic chemotherapeutic agents, INZ-C is highly hydrophobic and insoluble in water; thus, its half-life and bioavailability may have limitations.

To improve INZ-C's efficacy, half-life, and bioavailability, we developed a nanoparticle delivery system composed of a chitosan exterior that was loaded with cyclodextrin-INZ-C host/guest complex to enhance the efficacy and bioavailability of INZ-C. Dimethyl-β-CD was employed first to develop a host/guest complex with INZ-C and subsequently, this complex was loaded in a chitosan nanoparticle synthesized using a previously established method 27. The type of nanoparticle, either chitosan loaded with INZ-C alone or chitosan loaded with CD-INZ-C complexes, were then optimized. We then evaluated the role of the ratio of CD/INZ-C on the potency. Following development and optimization of the nanoparticles, we also evaluated the drug release, *in vitro* activity, the expression of p53 and its target gene-encoded proteins, and *in vivo* tumor reduction efficiency in a xenograft mouse model. As detailed below, our results demonstrate that this approach indeed improves the pharmacological effects and extends the *in vivo* half-life of INZ-C significantly.

## Methods

### Materials

Chitosan, heptakis (2,6-di-O-methyl)-β-cyclodextrin, tripolyphosphate (TPP), NaOH, and d4-DMSO, were purchased from Sigma Aldrich. Anti-p53 (DO1), and anti-PUMA (H-136) were purchased from Santa Cruz Biotechnology. Anti-p21 (CP74) was purchased from Neomarkers, Fremont, CA, USA. Cleaved PARP (catalog no. #9541) was purchased from Cell Signaling Technology. GAPDH (catalog no. 60004) was purchased from Proteintech. Dithiothreitol, TRIS base, sodium chloride, acetonitrile, glycine, sodium chloride, N'N Methylene Bisacrylamide, and Ethyl triacetic diamine acid (EDTA) were purchased from Fisher Scientific. Phenylmethylsulfonyl Fluoride (PMSF) was purchased from Amresco, Solon, OH, USA. Methylsulfoxide and acrylamide were purchased from Acros Organics. Tween 20 was purchased from Zacros America, Newwark, DE. Dulbecco's modified Eagle's medium, Fetal Bovine Serum and Penicillin/Streptomycin were purchased from Gibco.

### Synthesis and characterizations of n-INZ-C and nanoparticle control

Encapsulation of INZ-C in chitosan cyclodextrin nanoparticles was performed by modifying an established method by Jingou. J et al. 27. Briefly, INZ-C was dissolved in DMSO and cyclodextrin dissolved in water and the two solutions were mixed and stirred for 4 h at 40 ºC. Subsequently, the mixture was evaporated, and the pellet was washed several times with water to remove any un-complexed reactant. Subsequently, the CD-INZ-C complex was dissolved in 5 mL of 1 mg/mL chitosan solution, and the pH of the solution was titrated to 4.5 with NaOH. Then, 1 mL of 1 mg/mL tripolyphosphate (TPP) was added to the solution and the resulting mixture was stirred at 800 rpm for 2 h. The nanoparticles were then centrifuged at 15,000 rpm for 30 min and dried. Nanoparticle controls (NPC) were synthesized in a similar fashion without INZ-C. DM-β-CD was dissolved in 1 mg/mL of TPP solution and this solution was mixed with 5 mL of 1 mg/mL pH 4.5 chitosan solution at 800 rpm for 2 h. Nanoparticles were then obtained by centrifugation similar to the n-INZ-C.

Nanoparticles were characterized using Fourier Transform Infrared Spectroscopy (FTIR), Nuclear Magnetic Resonance (NMR), Dynamic Light Scattering (DLS) and Transmission Electron Light Microscopy (TEM). For FTIR measurements, powder nanoparticle was processed using a KBr press and then employed for FTIR analysis using the optical spectroscopy facility in Department of Chemistry at Tulane University. For NMR characterization, the complex of CD and INZ-C was dissolved in d4-DMSO and the resulting solution was assessed using NMR. For DLS and TEM measurements, the nanoparticles were resuspended in 0.22 micron filtered PBS buffer and spotted on a TEM grid for TEM analysis. For DLS and zeta potential measurements nanoparticles were centrifuged, lyophilized and suspended in PBS+ 10% serum for analysis. Suspended nanoparticles were also stored in -20 °C and measured 1 month following storage for evaluation of nanoparticle stability. Zeta potential measurements were collected after 183 seconds of equilibration.

### Encapsulation efficiency and loading capacity of n-INZ-C

A calibration curve was generated by dissolving INZ-C in DMSO, and the UV absorption at 340 nm (λ_max_ of INZ-C) was measured. Subsequently, following nanoparticle synthesis, the nanoparticles or any un-encapsulated INZ-C was removed by washing the nanoparticles pellet. Then, the nanoparticles were dissolved in DMSO (to release the INZ-C), and the absorption at 340 nm (λ_max_ of INZ-C) was compared to the absorption of the concentration initial INZ-C used for synthesis of the nanoparticle to determine the percentage encapsulated. Concentration was determined from absorbance based on the calibration curve presented in the supplemental information. A similar technique was used to determine the loading capacity of n-INZ-C which was calculated by determining the amount of INZ-C loaded and calculating the ratio of this to the total weight of the nanoparticle.

### Drug release studies

For the drug release studies, a solution of INZ-C nanoparticles was placed in a dialysis bag stirred in PBS + 10% serum for 24 h. The UV absorption of the outside PBS solution was measured at various time points, and the concentration was calculated based on a calibration curve of INZ-C.

### Cell culture

Human colorectal carcinoma HCT116 +/+ and HCT116 -/-, human lung non-small cell carcinoma H460 and H1299, human breast cancer MCF-7, human embryonic fibroblast Wi38, mouse embryonic fibroblast (MEF) cells, human normal fibroblast (NHF) and human melanoma cells (SKMEL 103, 147, 5, and 28) were all used for this study. The human melanoma cancer cell lines SK-Mel-103 and SK-Mel-147 were cultured as described by Jiang, L et.al 35. All cells were cultured in Dulbecco's modified eagles medium (DMEM) with 10% fetal bovine serum, and penicillin/streptomycin at 37 ºC and 5% CO_2_.

### Inhibition of cell proliferation

Cells were seeded in a 96-well plate (2500 cells/well) and allowed to attach overnight. Subsequently, cells were treated with 0.5 μM n-INZ-C, 0.5 μM INZ-C or the respective controls. An Incucyte system was used to monitor the cell proliferation of the cells for 3 days through imaging. Subsequently, the software was used to determine cell confluence from the images and plotted to assess the inhibition of cell proliferation.

### Inhibition of wound healing and trans-well migration assay

Cells were seeded in a 96-well plate to 80-90% confluence and allowed to attach overnight. A wound was then created in each well using a wound maker and the cells were treated with 0.5 μM n-INZ-C, 0.5 μM INZ-C or the respective controls. The wound width and confluence were monitored using an Incucyte imaging system for 2 days.

### Cellular uptake studies

Cells were seeded in a 6-well plate and allowed to attach overnight. Cells were then treated with either 12.5 μM INZ-C or 12.5 μM of n-INZ-C for overnight. Then, cells were washed with cold PBS 3X and incubated on ice with 200 μL of DMSO with constant vertexing. After 5 h, the solution was centrifuged for 20 s and the absorbance was measured at 340 nm. A control without treatment was always used to ensure no background absorbance at that wavelength. A calibration curve of the absorbance INZ in DMSO at 31.25, 62.5 and 125 μM was generated, and later used to extrapolate the amount of INZ internalized in the cell.

### Immunoblotting

Cells were seeded at 70% confluence in a 60 mm dish for 24 h. Subsequently, cells were treated with INZ-C, NPC, n-INZ-C, CD or DMSO for 18 h and harvested for immunoblotting (IB). Cells were lysed with 50 mM TRIS pH 8.0, 150 mM NaCl, 5 mM EDTA, 0.5% NP40 buffer supplemented with 2 mM DTT and 1 mM PSMF buffer. Protein concentration was then measured using the Bradford protein assay and SDS-PAGE was performed using 25 μg of protein. Subsequently, the SDS-PAGE was transferred to a PDF membrane for IB and all membranes were probed with primary antibodies overnight at 4 ºC and horseradish-peroxidase-conjugated secondary antibody for 1 h at RT, devolved using a chemiluminescence detection kit, and finally visualized by an Omega 12iC Molecular Image System (UltraLUM). IB was performed using p53 (DO1), cleaved PARP (catalog no. #9541), p21 (CP74), PUMA (H-136), and GAPDH (catalog no. 60004) antibodies.

### Animal experiments

#### PK studies in mice

All animal experiments were performed following an IAUCC approved protocol. For the pharmacokinetic (PK) studies, C57BL/6 mice bought from Jackson Laboratories were injected with 45 mg/kg of n-INZ-C and blood was drawn from the tail vein at several time points ranging from 0.5-8 h. An acetonitrile extraction method was used to extract the INZ-C from the plasma. An INZ-C analog previously developed in our lab was used as an internal standard. Firstly, the blood was centrifuged at 14000 rpm to separate the plasma. Subsequently, 20 µL of acetonitrile containing an internal standard 1-(10*H*-phenothiazin-10-yl)-2-([1,2,4]triazolo[1,5-*a*]pyrimidin-2-ylsulfanyl)ethanone) was added to 50 µL of plasma for each time point. The tube was then vigorously shaken and centrifuged for 30 seconds. The pellet was discarded, and the supernatant was used for HPLC detection of INZ-C. HPLC detection was done in aqueous solvent containing 3% acetonitrile. A calibration curve with concentrations ranging from 10-100 nM of INZ-C was also developed using this method. This calibration curve was then employed to determine the concentration of INZ-C in mice plasma.

#### Toxicity studies in mice

For the toxicity studies, NOD/SCID mice were injected (IP) twice daily with either 30 mg/kg of n-INZ-C and 50 mg/kg NPC, 25 mg/kg NPC or PBS for 7 days. The weight of mice was monitored twice/week. After 7 days, the mice tissues were harvested in formalin and further processed for H/E staining.

#### Xenograft model

For the H460 xenograft studies, ten-week old nude mice purchased from Jackson Laboratories were injected subcutaneously with 1.7 × 10^6^ H460 cells. A digital caliper was used to measure the tumor volume. Measurements were taken every other day, and tumor volume was calculated using the formula 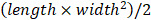. Following 7 days of injection of the cells, the mice were injected with either 50 mg/kg of nanoparticle control, or 30 mg/kg of INZ-C or of n-INZ-C. Tumor tissues were harvested and either snap-frozen in liquid nitrogen or stored in formalin following 18 days of drug treatment and endpoint tumor weights were also measured. Tissues snap-frozen in liquid nitrogen were further processed for immunoblot analysis. Tissues stored in formalin were further processed for H/E staining. All experimental protocols were approved by the Tulane University School of Medicine Institutional Animal Care and Use Committee.

## Results

### Synthesis and characterization of n-INZ-C

**Figure 1:**
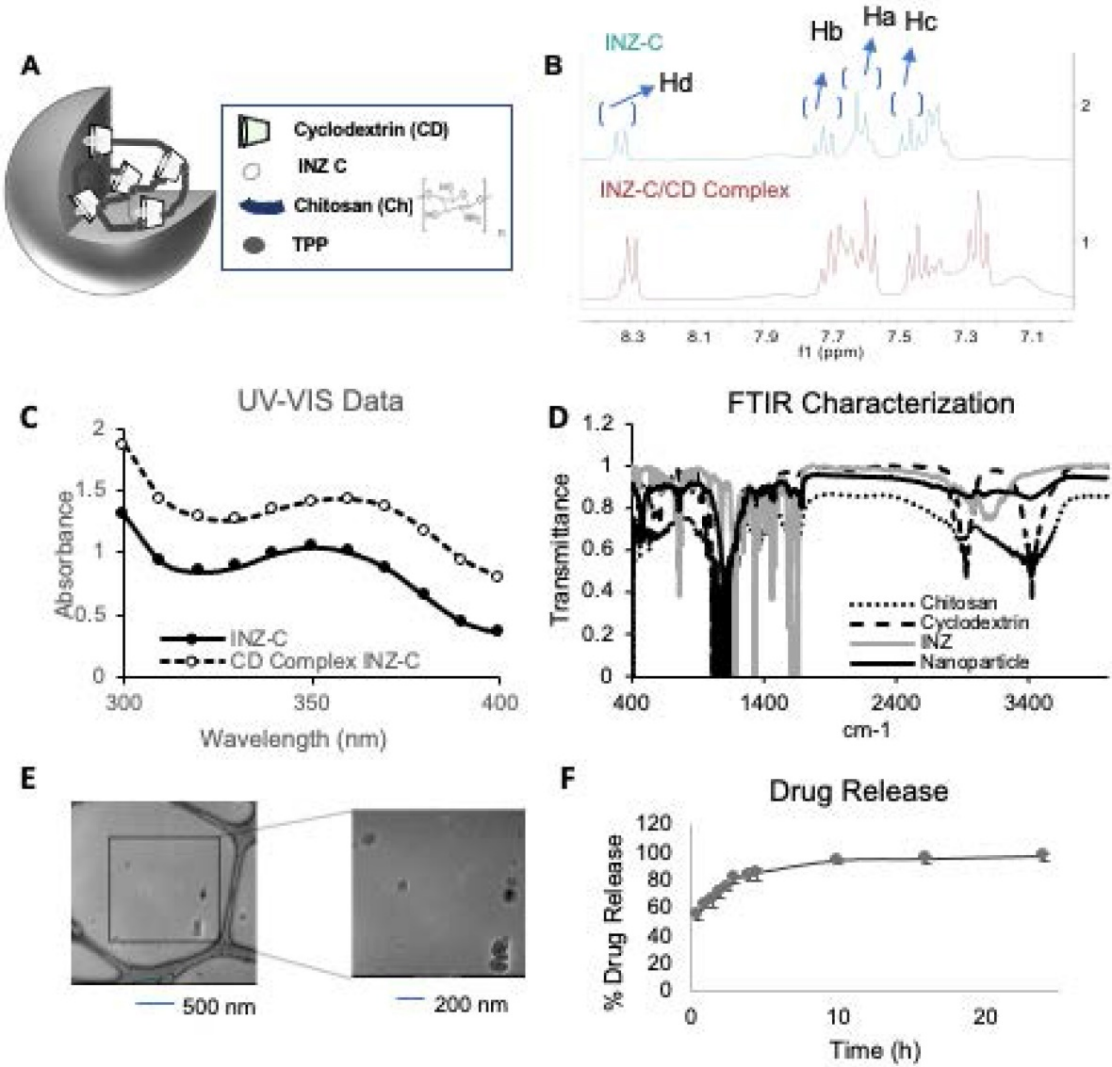
** Chemical characterization. (A)** Nanoparticle drug encapsulation structure. **(B)** NMR characterization of INZ-C and INZ-C/CD complex with peak identification of aromatic hydrogens. **(C)** UV Vis absorbance of INZ-C and INZ-C/CD complex. **(D)** FTIR characterization of chitosan, cyclodextrin, INZ-C, and n-INZ-C. **(E)** Cryo-Transmission Electron Microscopy (TEM) characterization of synthesized nanoparticles indicating a size of around 105 nm. **(F)** Drug release of nano-INZ-C over 24 h in PBS buffer + 10% serum (FBS).

To improve INZ-C's efficacy and half-life *in vivo*, we first synthesized INZ-C nanoparticles by using an established method for encapsulation of paclitaxel using chitosan and cyclodextrin (CD). A model for the nanoparticle is depicted in **Figure 1A**. The final synthesized product was characterized using nuclear magnetic resonance spectroscopy (NMR) and Fourier transform infrared spectroscopy (FTIR). NMR characterization was first used to confirm formation of the CD/INZ-C complex before encapsulation in the chitosan nanoparticles. As shown in **Figure 1B and S1**, shifting in several NMR peaks was observed with the complex formation as compared to the INZ-C and CD alone. As shown in **Figure 1B**, the up-field shift of the doublets observed at 8.32 ppm and 7.68 ppm along with the indole (NH) proton at 12.6 ppm were indicative of cyclodextrin encapsulation of the INZ-C. Additionally, as shown in **Figure 1C**, a red shift was observed in UV-Vis absorption spectra of the CD-INZ-C complex and the INZ-C alone. This, along with the increased hyperchromicity, is also suggestive of successful encapsulation of INZ-C within the CD cavity. FTIR characterization of the nanoparticle is presented in **Figure 1D**. Together, these results suggest that the final synthesized product contains INZ-C, chitosan and cyclodextrin combined as expected.

Formation of nanoparticles was then determined using dynamic light scattering (DLS), and transmission electron microscopy (TEM). As shown from the TEM Image and the DLS characterizations in **Figures 1E and S2A**, respectively, and the histogram in **Figure S3A**, TEM microscopy portrayed nanoparticles of 105 nm while DLS indicated formation of nanoparticles of around 150 nm. **Figure S2A** shows the DLS measurements of nanoparticles suspended in PBS + 10% serum immediately after synthesis and re-suspended after storage in -20 °C for a month. This study demonstrates that while a minor decrease in size is observed after 1 month, the nanoparticle size is relatively stable following long-term storage (up to a month) in -20 °C. Aggregation is not observed as no substantial shifting of the size distribution curve toward larger size is observed. Additionally, as shown in **Figure S2B**, the zeta potential of the nanoparticles was -10 mV for the sample suspended immediately after synthesis and -35 mV for the sample tested after a month of storage in -20 °C in PBS + 10% serum. A zeta potential of ±30 mV is indicative of stable nanoparticles since the large charge leads to an electrostatic repulsion between the nanoparticles and aids in their suspension. From our results, it seems the nanoparticles display stability following long term storage in PBS + 10% serum in -20 °C.

Following characterization of the chemical makeup and nanoparticle size of n-INZ-C, the drug encapsulation efficiency and release of this nanodelivery system were investigated. Characterization of nanoparticle drug encapsulation efficiency was evaluated by modifying a previously used technique and revealed a 30% drug encapsulation efficiency **(Figures S3B and S3C)** 27. The loading capacity was also calculated using a similar technique and was found to be 37%. Subsequently, the release of INZ-C from the nanoparticles in PBS solution (pH 7) + 10% serum was evaluated using a dialysis membrane. As shown in **Figure 1F**, more than 90% of the drug was released after 5 h. These findings were consistent with those presented in a previous investigation of chitosan/cyclodextrin nanoparticle encapsulation of paclitaxel 27. Together, these results indicate that we have successfully generated nanoparticles-encapsulated and effectively released INZ-C.

### *In vitro* activity of n-INZ-C

Along with this detailed characterization of the drug delivery system, the p53 induction and inhibition of cell proliferation following 18 h treatment of colorectal cancer HCT116^p53+/+^ cells with different doses of INZ-C, nanoparticle, or n-INZ-C was evaluated. Firstly, the effect of chitosan nanoparticles loaded with INZ-C alone or CD-INZ-C complex was investigated. Since the nanoparticles loaded with the CD-INZ-C complex displayed better p53 activation, the effect of the ratio of INZ-C and CD in the activity of n-INZ-C was then evaluated by testing the potency of different ratios (1:20, 1:15 and 1:10) of INZ-C and CD on p53 activation. Our results demonstrated that 0.5 μM of 1:20 ratio (INZ-C/ CD) was the most optimal for enhanced activity of n-INZ-C; thus, this ratio and concentration was employed for all further studies, **(Figures S4A and S4B)**. Stability of the n-INZ-C was also evaluated after storage for 1 month in PBS buffer **(Figure S4C)**, and minimal difference was observed in activity suggesting a good stability.

**Figure 2:**
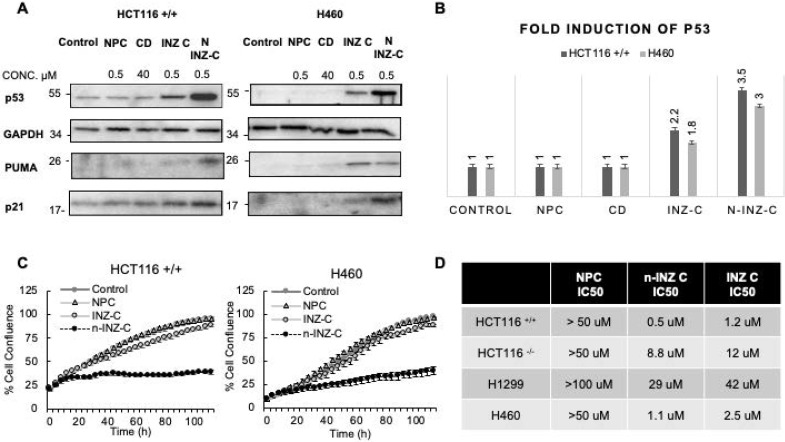
***In-vitro* activity. (A)** IB of HCT116 +/+ and H460 cells after treatment with various controls, INZ-C and n-INZ-C for 18 h. **(B)** Quantification of the IB presented in figure 2a. **(C)** Cell proliferation assay of HCT116 p53 +/+ and H460 cells after treatment with PBS control, NPC, 0.5 μM INZ-C, 0.5 μM n-INZ-C treatment for 3 days. **(D)** IC_50_ table of NPC, INZ-C, and n-INZ-C in HCT116 ^+/+^, HCT116 ^-/-^, H460 and H1299 cells.

The result from the IB analysis **(Figure 2A)** indicated that p53 is more significantly induced in HCT116 +/+ colon cancer and H460 (p53 wt) lung cancer cells by n-INZ-C than that by INZ-C only, following 18 h treatment with DMSO (control), CD, and nanoparticle control (NPC), INZ-C or n-INZ-C. Quantification of the IB bands revealed about a 2-fold induction of p53 by INZ C in both cell lines, while about a 3-3.5-fold induction was observed for the n-INZ-C **(Figure 2B)**. In addition to induction of p53, p53 target genes such as PUMA and p21 were also induced with INZ-C and n-INZ-C treatment. However, similar to the enhanced p53 induction, induction of these target genes was more enhanced following nanoparticle encapsulation of n-INZ-C. This induction was p53-dependent, as it was not evident in p53 null HCT116 and H1299 cells **(Figure S5A)**. More drastic activation of the p53 pathway by n-INZ-C than that by INZ-C only was also evident in melanoma and breast cancer cells **(Figure S6A)**. These results indicate that n-INZ-C is more effective than INZ-C alone in the induction of the p53 pathway in cancer cells.

In order to corroborate the enhanced p53 activation with *in vitro* activity, the effect of n-INZ-C on the inhibition of cell proliferation was then investigated. As shown in **Figure 2C**, when p53-containing HCT116 and H460 cells were treated with or without 0.5 µM of INZ-C or n-INZ-C for 4 days, their proliferation was more markedly inhibited by n-INZ-C than INZ-C alone at this low dose. This result was also reproduced in other p53-containing cancer cells, such melanoma and breast cancer cells **(Figures S6A and S6B)**. Although this inhibitory effect was less dramatic in p53-null lung and colorectal cancer cells, it was evident in these cells **(Figure S5B)**, suggesting that n-INZ-C might possess some p53-independent activity against cancer cells, which is consistent with our previous observation 4. Dose dependent inhibition of cell proliferation was then investigated to determine the IC_50_ concentration. As shown in **Figure 2D**, the IC_50_ concentration of INZ-C was reduced by two-fold following nanoparticle encapsulation, which further corroborates the enhanced p53 induction and improved inhibition of proliferation observed with n-INZ-C. All individual IC_50_ curves are presented in **Figures S7A-D**. Thus, these results demonstrate the markedly improved activity of the INZ-C following nanoparticle encapsulation.

**Figure 3:**
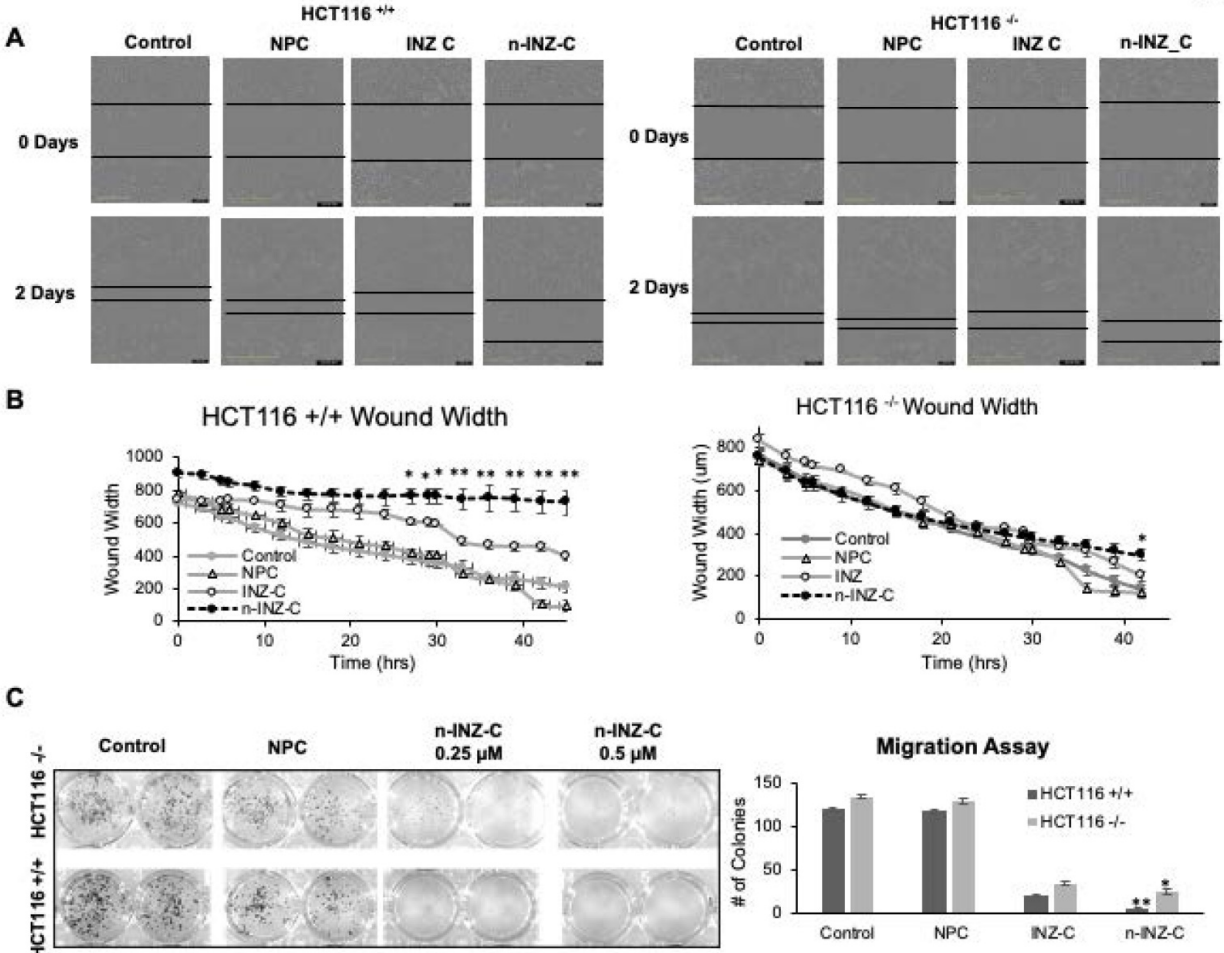
** Wound healing and migration assay. (A)** Picture representations of wound healing assay for HCT116 +/+ and HCT116 -/- cell lines before and after 3 day treatment of INZ and n-INZ-C. **(B)** Quantification of wound width for the wound healing assay following 2 day treatment of INZ and n-INZ-C. * p ≤ 0.05 and ** p ≤ 0.01 where n=4. **(C)** Trans-well migration assay for HCT116 +/+ and -/- cell lines following 5 day treatment of 0.25 and 0.5 µM of n-INZ C * p ≤ 0.05 and ** p ≤ 0.01 where n=4.

Next, we compared n-INZ-C with INZ-C in their effects on the wound healing and cell migration of colorectal cancer cells. As shown in **Figure 3A**, a larger wound width was observed at the endpoint for HCT116 ^p53+/+^ cells treated with INZ-C or n-INZ-C as compared to the DMSO and nanoparticle controls. In addition, the wound width for n-INZ-C was significantly (p ≤ 0.005 where n=4) larger than that of INZ-C **(Figure 3B)**. Consistent with the results from the wound healing assay, results from our trans-well migration assay also demonstrate a significant reduction in the number of cells migrated to the 24 well plate following 5-day treatment of n-INZ-C as compared to INZ-C **(Figure 3C)**. Intriguingly, HCT116^p53-/-^ cells also demonstrated some degree inhibition of wound healing and cell migration with n-INZ-C and INZ-C treatment suggesting some p53 independent activity. However, comparison of the change in wound width of HCT116^p53-/-^ and HCT116^p53+/+^ cells following treatment of n-INZ-C indicates a 4-fold larger change in wound width in the HCT116^p53+/+^ cells. This suggests that while this mechanism could be partially p53 independent, it seems to be predominantly p53 dependent. Further studies will be needed to dissect the underlying mechanisms; however, from these results, we can conclude that in addition to enhancing the p53 induction and inhibition of cell proliferation, the nanoparticle encapsulation also results in enhanced inhibition of wound healing and migration properties.

### *In vitro* and *in vivo* toxicity of n-INZ-C

**Figure 4:**
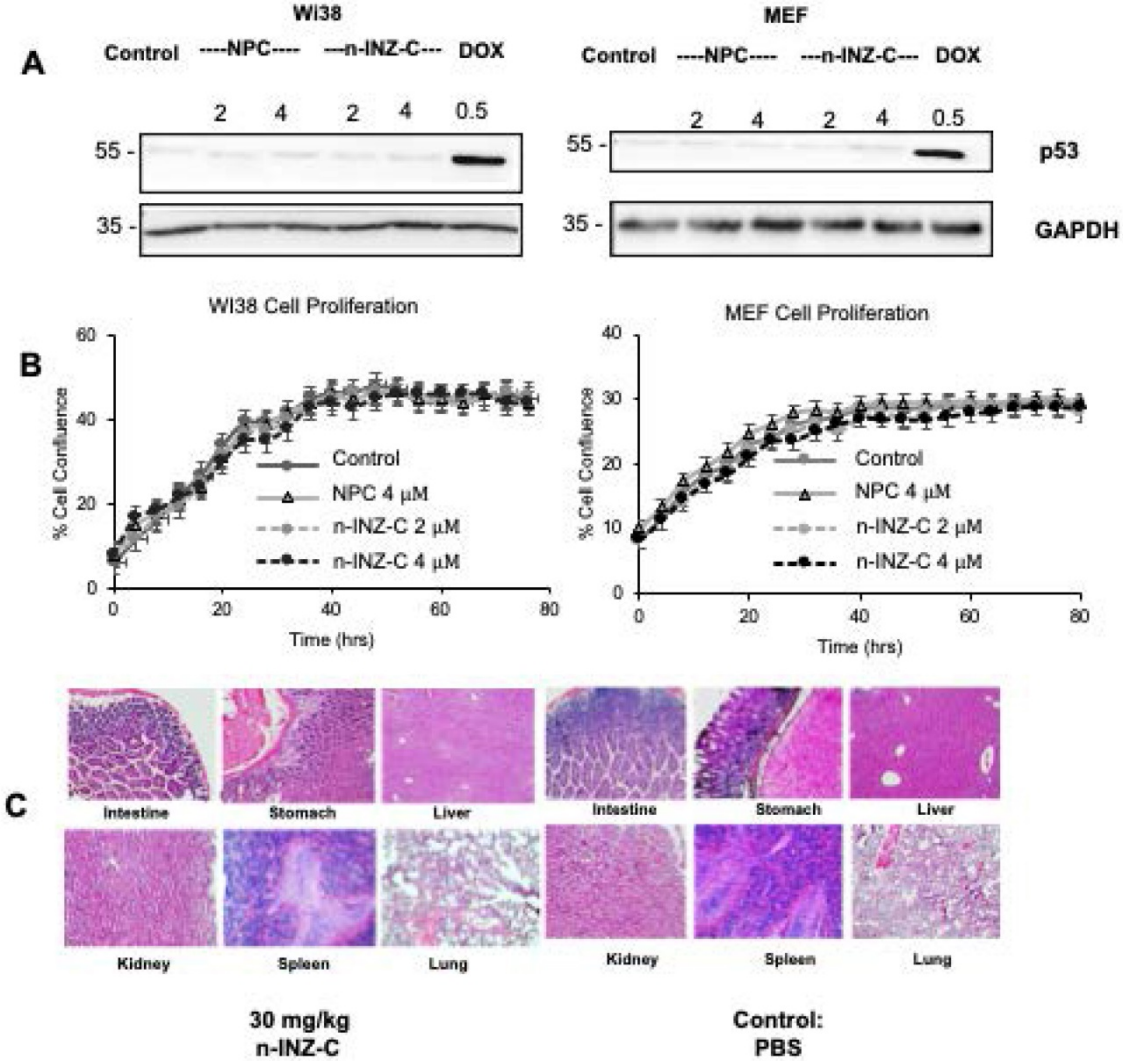
***In vitro* and *in vivo* toxicity of n-INZ-C. (A)** No p53 induction in Wi38 and MEF cells with n-INZ C, INZ-C and nanoparticle control treatment. **(B)** Inhibition of cell proliferation with n-INZ-C, INZ-C and NPC treatment in Wi38 and MEF cells. **(C)** H/E staining of tissues harvested after mice were injected for 7 days with 30 mg/kg n-INZ-C or PBS.

As the results from cell proliferation, wound healing and migration assays demonstrated the strong therapeutic potential of n-INZ-C, the toxicity of the nanodrug delivery system was then evaluated both *in vitro* and *in vivo*. In this regard, normal human fibroblast Wi38 and murine embryonic fibroblast (MEF) cells were used to assess the *in vitro* toxicity of the nanodrug. As shown in **Figure 4A**, no p53 induction was observed after either INZ-C or n-INZ-C treatment in both cell lines. Furthermore, as shown in **Figure 4B**, no difference in the inhibition of cell proliferation as compared to the controls was observed after INZ-C or n-INZ-C treatment in either cell line suggesting minimal to no toxicity of this drug. To further confirm these results, toxicity of n-INZ-C treatment was then evaluated *in vivo*, as toxicity of INZ-C alone has already been previously evaluated and published in our lab 36. **Figure 4C** presents the H/E staining of tissues from mice following n-INZ-C or PBS control treatment for 7 days. H/E staining of tissues from mice treated with a high and low dose of nanoparticle control is presented in **Figure S8A**. The weight of the mice during the 7 day treatment is depicted in **Figure S8B**.These results were corroborated with our pathology expert to ensure proper interpretation as well. From these results, we can conclude that n-INZ-C displays minimal toxicity both *in vitro* and *in vivo*, as all of the H/E staining displayed no apparent pathological alternations, and the body weights of the mice in the all of the groups did not show any difference **(Figures 4C, S8A, and S8B)**.

### Enhanced bioavailability of n-INZ-C

**Figure 5:**
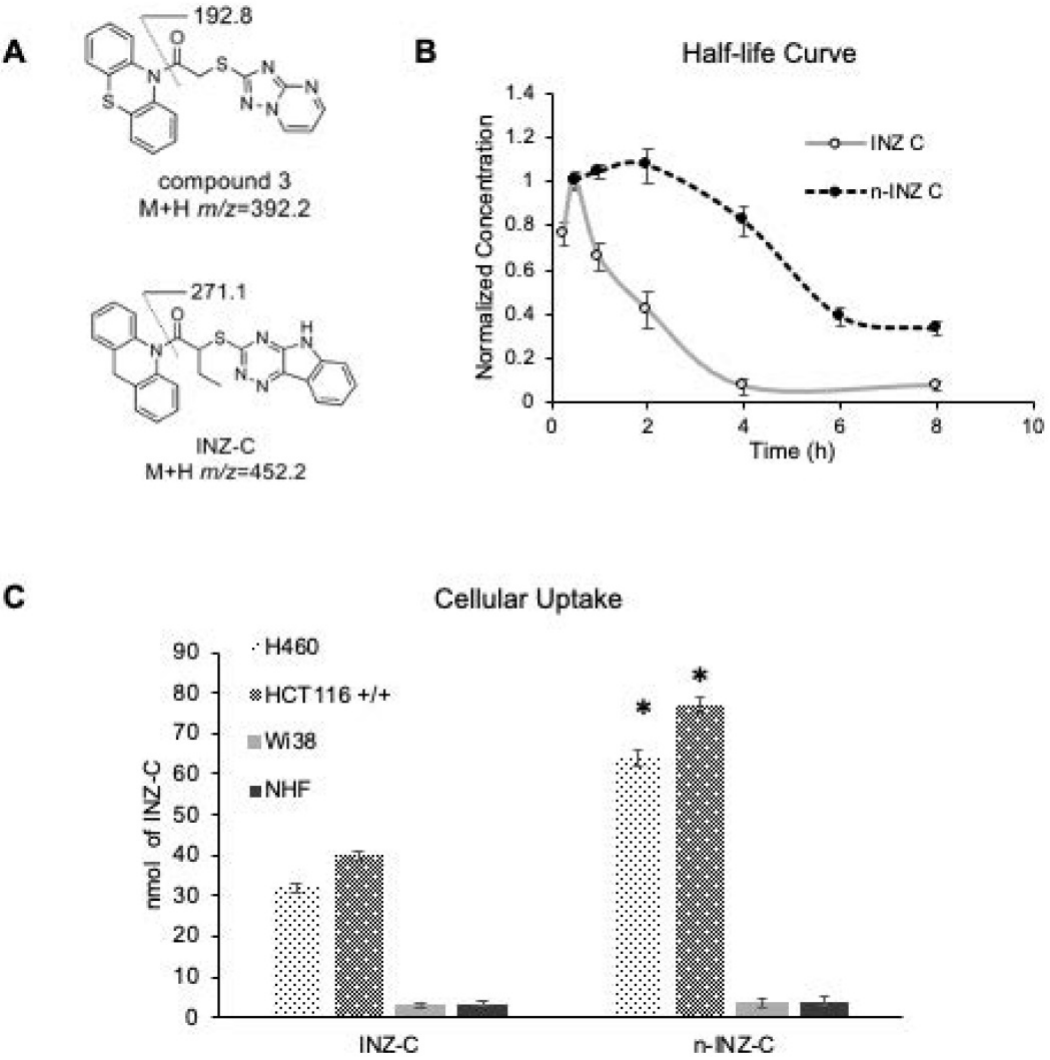
** Pharmacokinetic studies of n-INZ-C. (A)** Structure of internal standard (1-(10*H*-phenothiazin-10-yl)-2-([1,2,4]triazolo[1,5-*a*]pyrimidin-2-ylsulfanyl)ethanone) and INZ-C. **(B)** Pharmacokinetic curve of n-INZ-C and INZ-C in mice plasma after 8 h with highest concentration normalized to 1. **(C)** Cellular uptake of n-INZ-C and INZ-C in H460, HCT116 +/+, WI38 and normal human fibroblast (NHF) cells.

High performance liquid chromatography (HPLC) was then employed for determination of the half-life of n-INZ-C in mouse serum. Mice were injected with n-INZ-C and blood was drawn at various time points and processed for HPLC analysis as described in the materials and methods. A calibration curve was first generated using HPLC to detect n-INZ-C concentration in FBS serum using an internal standard method using the internal standard (1-(10*H*-phenothiazin-10-yl)-2-([1,2,4]triazolo[1,5-*a*]pyrimidin-2-ylsulfanyl)ethanone) **(Figures 5A and S9)**. Subsequently, this calibration curve was used to determine the n-INZ-C concentration in serum collected at 0.5, 1, 2, 4, 6 and 8 h from mice injected with 45 mg/kg of n-INZ-C. The results were then compared to the half-life curve determined for INZ-C. As shown in **Figure 5B**, nanoparticle encapsulation doubled the half-life of INZ-C. Additionally, cellular uptake studies *in vitro* displayed enhanced cellular uptake of the INZ-C as well **(Figure 5C)** further corroborating these results. Similar to the INZ-C without nanoparticle encapsulation, minimal internalization was observed in normal cells for n-INZ-C.

### *In vivo* tumor reduction with n-INZ-C treatment

**Figure 6:**
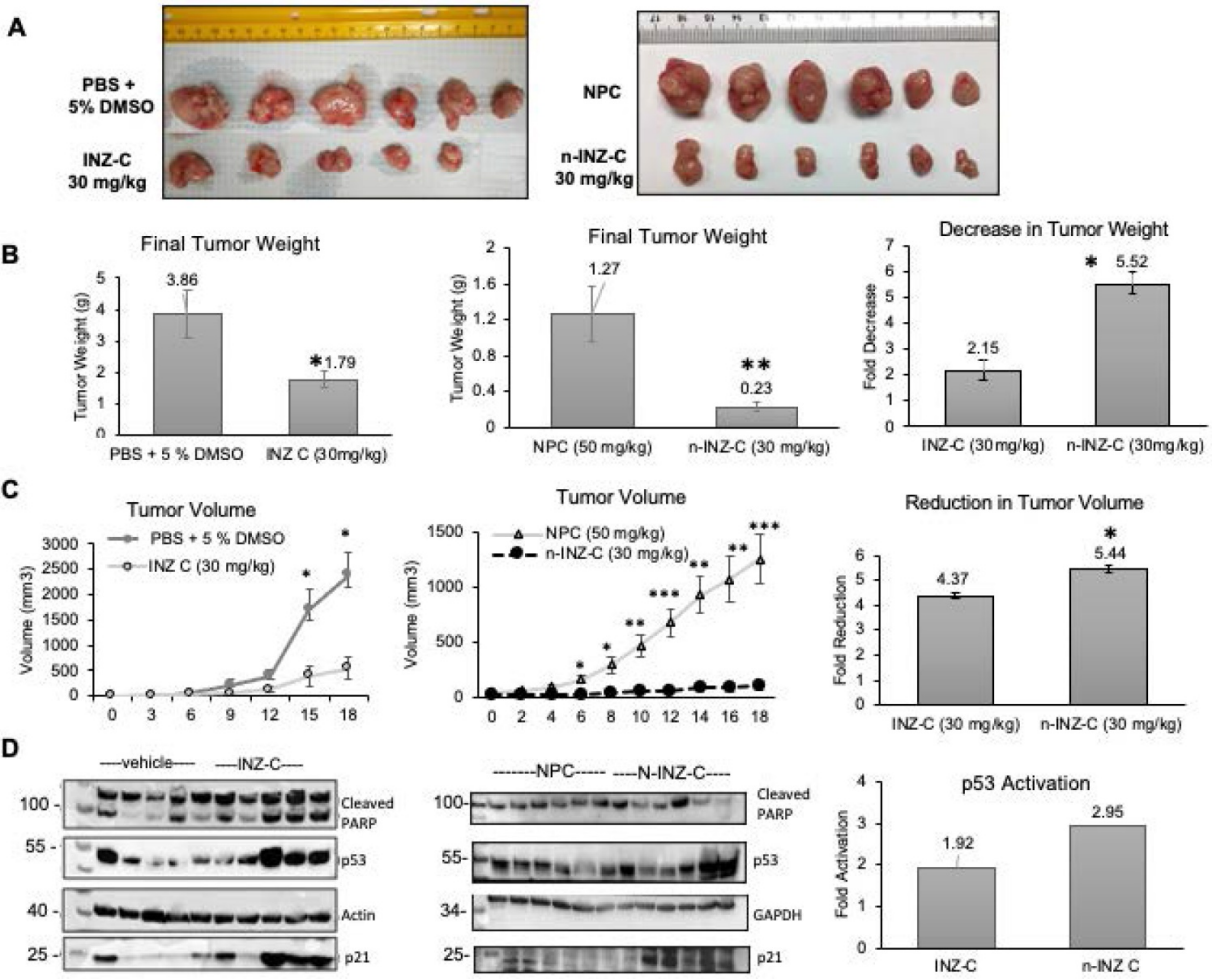
** H460 xenograft tumor model. (A)** Picture representation of tumors after 18-day drug treatment. **(B)** Reduction in tumor weight following 18-day treatment of PBS+ 5 % DMSO, INZ C, nanoparticle control (NPC), and INZ-C nanoparticle (n-INZ-C) * p ≤ 0.05 and ** p ≤ 0.005 where n=6. **(C)** Reduction in tumor weight following 18-day treatment of PBS + 5% DMSO, INZ C, nanoparticle control (NPC), and INZC nanoparticle (n-INZ-C). * p ≤ 0.05 and ** p ≤ 0.005 where n=6. **(D)** P53 induction of tumor tissues following 18-day treatment of PBS + 5% DMSO, INZ C, nanoparticle control (NPC), and INZC nanoparticle (n-INZ-C) where n=6.

In order to evaluate the *in vivo* activity of n-INZ-C, we assessed the inhibition of tumor growth in a H460 xenograft tumor model. **Figure 6A** is a picture representation of the end-point tumors from the H460 xenograft model. As shown by the final tumor weights **(Figure 6B)** for the mice as presented in **Figure 6B**, n-INZ-C repressed the growth of the xenograft lung tumors by 5.5-fold in comparison with the tumors treated with NPC, while INZ-C only reduced it by ~2-fold at the same dose as that used for n-INZ-C. Additionally, as shown in **Figure 6C**, a statistically significant difference in tumor volume as compared with the control was observed for the n-INZ-C treated mice starting from day 6 after treatment. In contrast, a significant difference in tumor volume of mice treated with INZ-C as compared to the control was only observed after 15 days of treatment. Evaluation of the p53 and p53 target gene expression using IB is displayed in **Figure 6D**. Intriguingly, enhanced p53 and p21 induction were observed in tissues from mice treated with n-INZ-C or INZ-C alone as compared to the mice treated with NPC. Throughout this experiment, there were no changes in mice's body weights **(Figure S10A)**. Also, the regressed xenograft tumors were evident in the H/E staining of the tumor tissues **(Figure S10B)**. Hence, these results indicate that n-INZ-C more effectively inhibits the growth of xenograft lung tumors than INZ-C alone.

## Discussion and Conclusion

From these results, we demonstrate substantial improvements in the *in vitro* and *in vivo* activity of INZ-C following encapsulation with the chitosan/CD nanocarrier. Our detailed characterization confirms the encapsulation of INZ-C within the nanoparticle (Figure 1). Upfield shifts in the aromatic hydrogens following INZ-C/CD complexation in our NMR characterization confirms encapsulation of INZ-C within the cavity of CD **(Figures 1B and S1)**. This is consistent with previous studies that demonstrated upfield shifting of guest molecule hydrogens with CD encapsulation 37-40. Furthermore, the red shift observed in absorbance (to longer wavelength) for our studies further confirms this CD encapsulation. Previous studies have attributed this red shift in absorbance to change in environment of the guest molecule following CD encapsulation. In this regard, the guest molecule is encapsulated in the nonpolar cavity of β-CD in contrast to the polar aqueous environment in the case of the free guest molecule 41. Moreover, from these results we can conclude that the CD has formed a host/guest complex with INZ, and this host/guest complex has been encapsulated within the chitosan nanoparticles observed in the images obtained from TEM as well as DLS characterization **(Figure 1E and S2)**. Stability of the nanoparticles was confirmed from the zeta potential and DLS measurements presented in Figure S2. Drug release studies also confirmed the release of the drug from the nanoparticle in PBS pH 7.4 + 10% serum.

Our* in vitro* investigation suggests a significant improvement of INZ-C with nanoparticle encapsulation. p53 induction and IC_50_ concentrations were improved by 2-fold as depicted by dose dependent cell proliferation charts as well as IB of p53 and p53 target genes in several cell lines following drug treatment, while minimal effect was observed in normal cells **(Figures 2 and 4)**. These results are consistent with many nanomedicine studies that attribute enhanced internalization of nanoparticles into tumors to the enhanced permeability and retention phenomenon 42, 43. In this regard, the leaky tumor vasculature allows for enhanced permeation of nanoparticles into tumors cells, while the rigid vasculature of normal cells prevents the rapid internalization into normal cells 42, 44, 45. The 2-fold enhancement in the cellular uptake in lung and cancer cells **(Figure 5)** suggests that this improvement in p53 induction is also probably attributed to the enhanced bioavailability. Consistent with the minimal activity, minimal internalization of INZ-C and n-INZ-C was observed in normal cells as depicted in the cellular uptake analysis **(Figure 5)**. In addition to enhanced *in vitro* bioavailability, the 2-fold enhancement in the half-life confirms that enhanced bioavailability is most probably observed *in vivo* as well **(Figure 5)**. This enhanced *in vivo* bioavailability is further corroborated by the 2-fold enhancement in tumor reduction in our xenograft model **(Figure 6)**. Additionally, consistent with the minimal activity and cellular internalization observed *in vitro,* the *in vivo* toxicity studies further confirmed the promising biocompatibility of both INZ-C and n-INZ-C **(Figure 4)**.

Interestingly, we have also observed the anti-metastatic effect of n-INZ-C or INZ-C **(Figure 3)**. This is the first time for us to discover this activity for the INZ family. Interestingly, this activity was also found in p53-null lung and colorectal cancer cells **(Figure 3)**. This finding suggests that the anti-metastatic effect of INZ-C might be independent of p53, yet its underlying mechanism remains unclear.

Moreover, we demonstrate that the n-INZ-C has improved the cellular uptake and half-life of INZ-C with reduced toxicity and display the properties of wound healing and of suppressing tumor growth *in vivo* as well. In this regard, the improved cellular uptake has been a common advantage of nanoparticle drug delivery systems due to the enhanced permeation and retention effect. This improved cellular uptake will in turn most likely enhance the efficacy of the drug as we observed here. This improved bioavailability was also confirmed from the improved *in vivo* ½ life of INZ that is observed with the nanoparticle encapsulation. All of these factors contribute to the improved *in vivo* tumor reduction as shown in **Figure 6**.

These results provide an insight to development of a nanodrug delivery for INZ-C that demonstrates promising *in vitro* and *in vivo* activity that may be employed for other lipophilic drugs as well. Additionally, the nanodrug also demonstrated mild activity in p53 null cell lines; thus, additional studies on p53 independent targets of this nanodrug system will further its application as well, such as via p73 4. Furthermore, the versatility of CD and chitosan in this nanodrug delivery system can be employed to further modify this system as needed to enhance the activity of other chemotherapeutic agents. Lastly, evaluation the synergistic effect of INZ with other chemotherapeutic agents following co-drug delivery using this system will provide further insights to its bio-applications.

## Supplementary Material

Supplementary figures and tables.
