# Supplementary material for: Nanoparticle encapsulation of non-genotoxic p53 activator Inauhzin-C for improved therapeutic efficacy
Source: Theranostics. 2021 May 12;11(14):7005–17. doi: 10.7150/thno.57404 (PMC8171090; doi:10.7150/thno.57404)
Supplement: Supplementary file 1 — Supplementary figures and tables. [file thnov11p7005s1.pdf]

Supplemental Information:

Nanoparticle Encapsulation of Non-genotoxic p53 Activator Inauhzin-C for Improved Therapeutic Efficacy

Nimisha Bhattarai[1], Jieqiong Wang[1], Daniel Nguyen[1], Xiaoxiao Yang[2], Linh Helmers[3], Jennifer Paruch[3,] Li Li[3] , Yiwei Zhang[1], Kun Meng [4], Alun Wang[5], Janarthanan Jayawikramarajah[4], Binghe Wang[2], Shelya Zeng[1*], and Hua Lu[1*]

Figure S1. NMR characterization of n-INZ-C

A. Structure of INZ-C molecule along with the identification of hydrogens for the NMR peaks presented in Figure 1B
B. NMR spectra of Heptakis(2,6-di-O-methyl)-β-cyclodextrin
C. NMR spectra of Inauhzin-C (INZ-C)
D. NMR spectra of the complex of INZ-C and CD

Figure S2. DLS and Zeta Potential of n-INZ-C

A. DLS of nanoparticles immediately following synthesis and after 1 month of storage in -20°C
B. Zeta potential of the nanoparticles immediately following synthesis and after 1 month of storage in -20°C

Figure S3. TEM histogram and UV-VIS Calibration

A. Histogram of nanoparticle sizes from TEM

B. UV Vis absorbance of INZ-C standards at different concentrations

C. Calibration curve generated from absorbance measurements in Figure S3B

Figure S4. Optimization of p53 activation by n-INZ-C

A. IB of HCT116$^{p53+/+}$ cells treated for 18 h. with n-INZ-C containing different ratios of CD/INZ-C ranging from 1:10, 1:15, and 1:20.

B. IB of dose dependent p53 activation of n-INZ-C. Cells were treated with either 1, 0.5 and 0.1 μM of n-INZ-C or INZ-C and the p53 and p21 activation was compared.

C. IB of cells treated with 0.5 μM of n-INZ-C initially and after 1 month of storage of n-INZ-C in -20ºC.

Figure S5. *In Vitro* activity of n-INZ-C in colon and lung cancer cell lines

A. IB of HCT116$^{p53-/-}$ and H1299 cells treated with 0.5 μM of INZ-C, n-INZ-C, CD, NPC and DMSO control for 18 h. IB was probed for p53, p21 and PUMA.

B. Cell proliferation of HCT116$^{p53-/-}$ and H1299 cells treated with 0.5 μM INZ-C, n-INZ-C, DMSO control and NPC for 3 days. Images were taken using incucyte and cell proliferation was calculated using incucyte S3 software. Data represents the average of triplicate measurements of the cell confluence.

C. Images taken using Incucyte of HCT116$^{p53+/+}$ and H460 cells after 68 hrs of treatment with 0.5 μM INZ-C, n-INZ-C, DMSO control and NPC.

Figure S6. *In vitro* activity in melanoma and breast cancer cell lines

A. IB of SKMEL 103, SKMEL 147 and MCF7 cells treated with with 0.5 μM of INZ-C, n-INZ-C, CD, NPC and DMSO control. IB was probed with p53, p21 and PUMA.

B. Cell proliferation of SKMEL 103, SKMEL 147 and MCF7 cells treated with INZ-C, n-INZ-C, NPC and DMSO control. All measurements of cell confluence were calculated from incucyte images using the incucyte S3 software. Results represent the average of triplicate measurements of cell confluence.

Figure S7. IC_{50} curves for NPC, INZ-C and n-INZ-C in colorectal and lung cancer cell lines

Dose dependent cell proliferation of NPC, INZ-C and n-INZ-C in (A) $HCT116^{p53+/+}$, (B) H460, (C) $HCT116^{p53-/-}$, (D) H1299. Measurements of cell confluence were calculated from incucyte images using the incucyte S3 software. IC_{50} was determined using the equation provided in materials and methods.

Figure S8. Mice weight and organ staining images from *in vivo* toxicity studies

A. H/E staining images of liver, kidney, spleen and lungs of mice treated with high dose (50 mg/kg) and low dose (25 mg/kg) of NPC for 7 days.

B. Weight of mice treated with n-INZ-C, high (50 mg/kg) and low dose NPC (25 mg/kg) and PBS control for 7 days

Figure S9. Calibration data for *in vivo* pharmacokinetic analysis

A. Peak areas of internal standard and n-INZ-C in samples of mice blood determined from MS analysis. Protein in blood samples was precipitated using acetonitrile precipitation as explained in the materials and methods. Samples were then diluted and subjected for MS analysis.
B. Calibration curve for MS detection of INZ-C in plasma data presented in Figure S9A.

Figure S10. Mice weight and tumor imaging from H460 Xenograft model

A. Mice weight during 18-day treatment with 30 mg/kg of n-INZ-C and NPC and 30 mg/kg of INZ-C and PBS + 5% DMSO control.
B. Images of tumors from mice treated with 30 mg/kg NPC and n-INZ-C

Figure S1

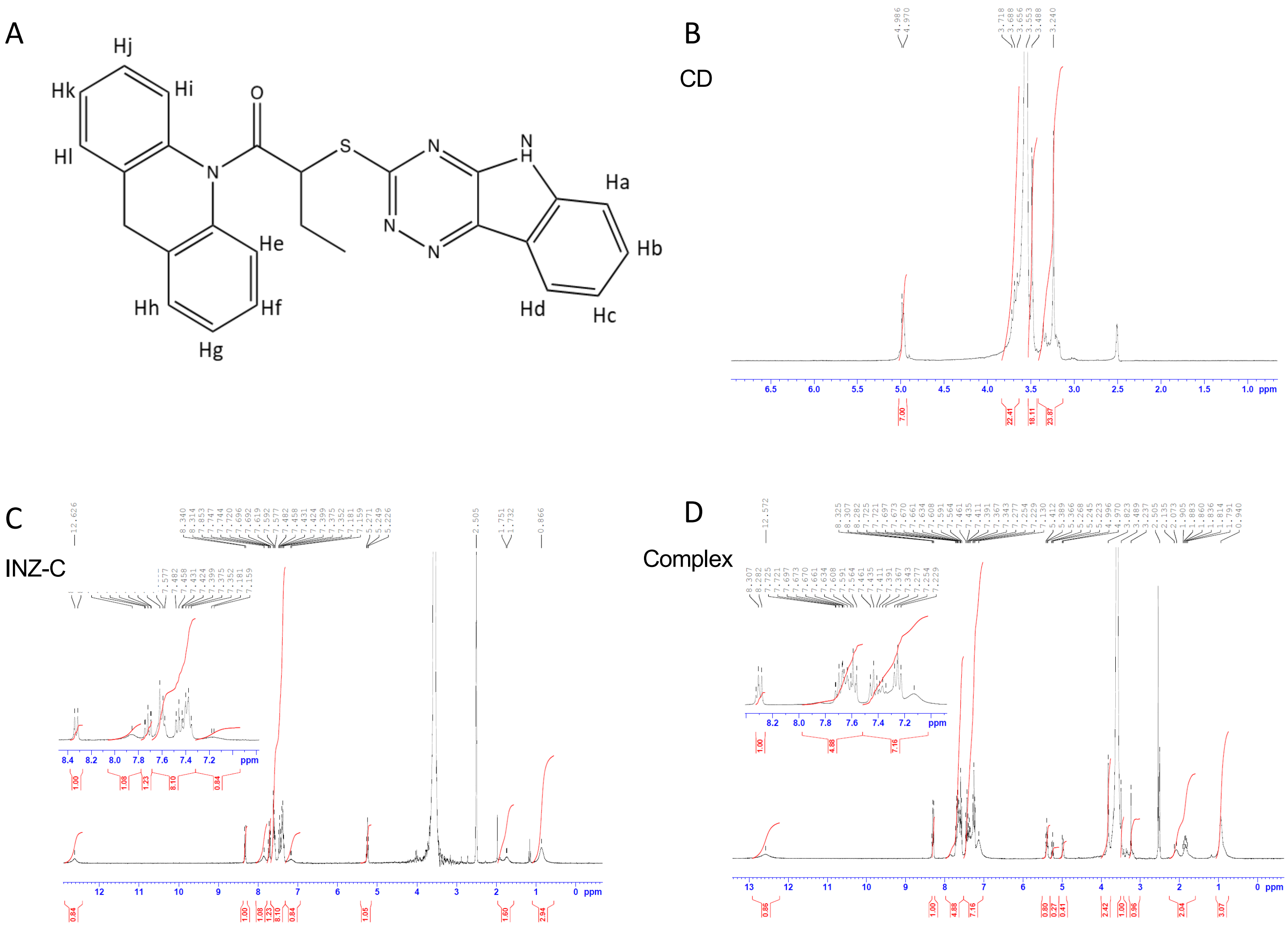

Figure S2

A

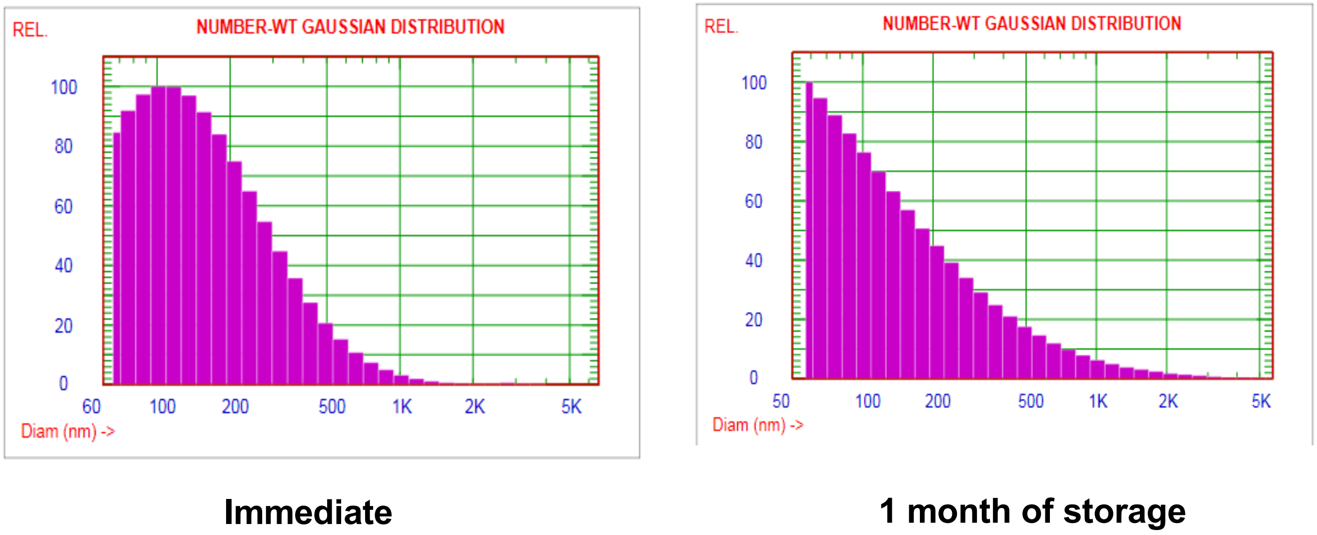

B

	Immediately after Synthesis	After 1 month storage
Zeta Potential (mV)	-10 mV	-35 mV

Figure S3

A

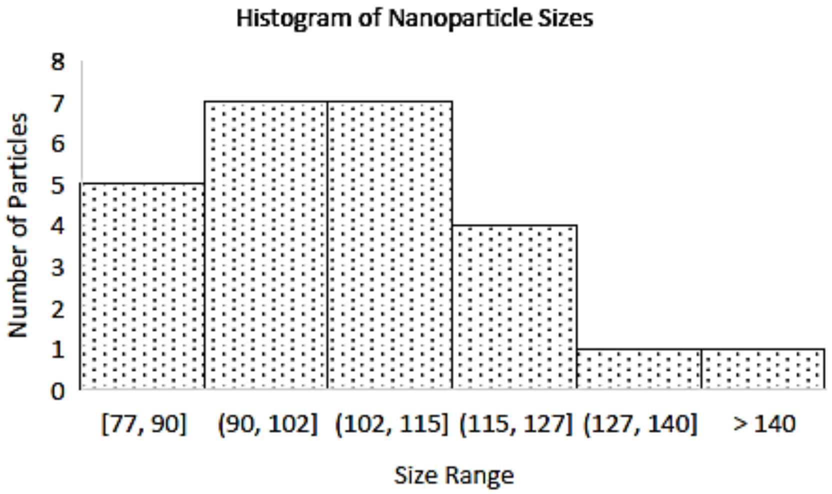

B

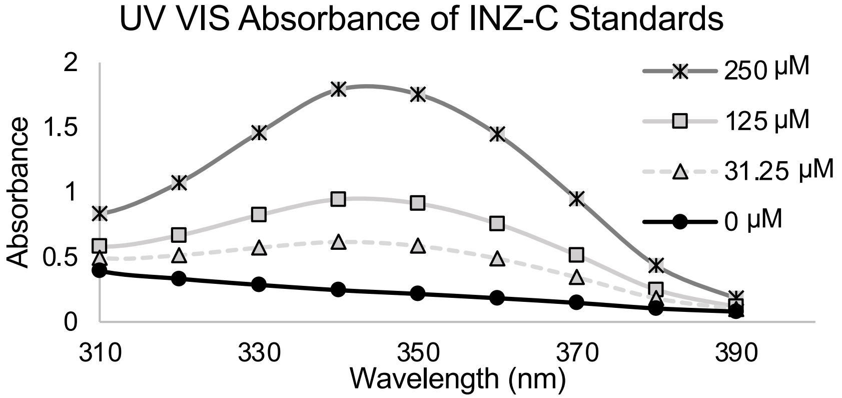

C

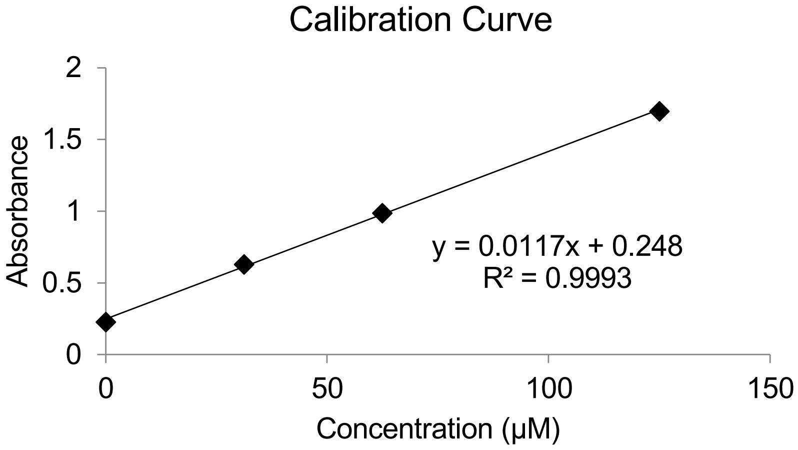

Figure S4

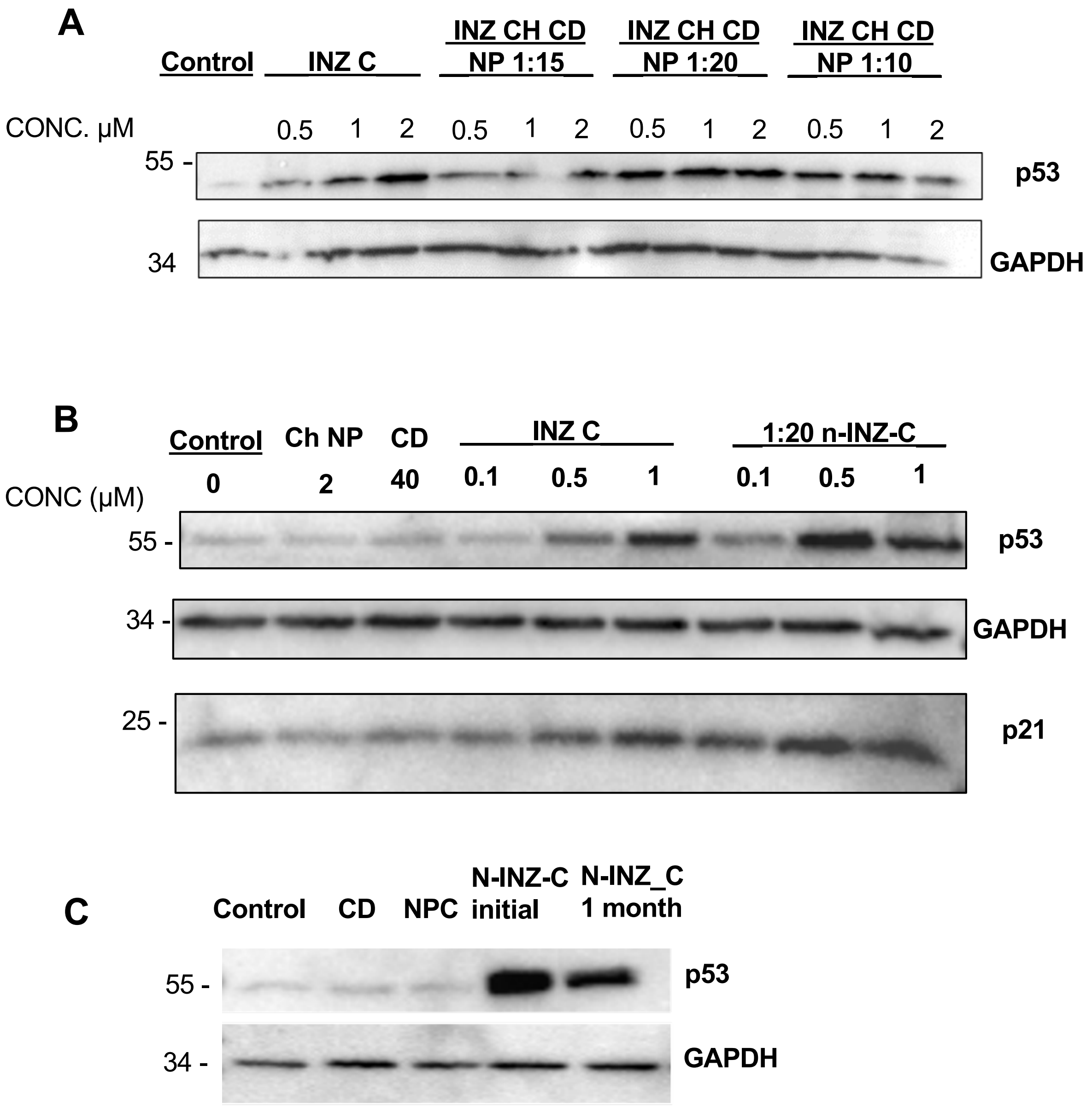
A
Control
INZ C
INZ CH CD NP 1:15
INZ CH CD NP 1:20
INZ CH CD NP 1:10
CONC. μM
0.5 1 2 0.5 1 2 0.5 1 2 0.5 1 2
55 -
p53
34
GAPDH
B
Control
Ch NP
CD
INZ C
1:20 n-INZ-C
CONC (μM)
0 2 40 0.1 0.5 1 0.1 0.5 1
55 -
p53
34 -
GAPDH
25 -
p21
C
Control
CD
NPC
N-INZ-C initial
N-INZ_C 1 month
55 -
p53
34 -
GAPDH

Figure S5

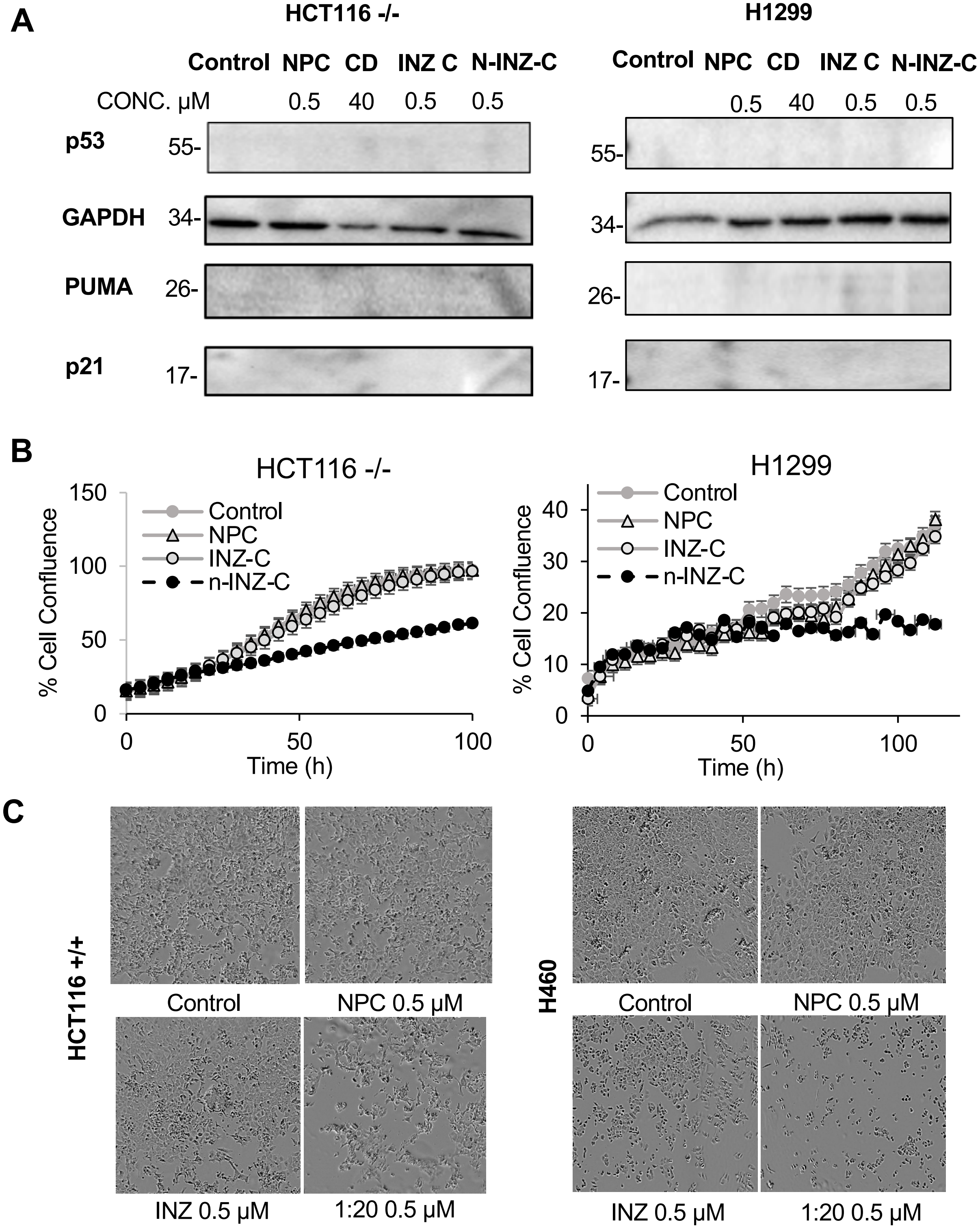

A
HCT116 -/-
Control NPC CD INZ C N-INZ-C
CONC. μM 0.5 40 0.5 0.5
p53 55-
GAPDH 34-
PUMA 26-
p21 17-
H1299
Control NPC CD INZ C N-INZ-C
0.5 40 0.5 0.5
55-
34-
26-
17-
B
HCT116 -/-
% Cell Confluence
Time (h)
Control
NPC
INZ-C
n-INZ-C
H1299
% Cell Confluence
Time (h)
Control
NPC
INZ-C
n-INZ-C
C
HCT116 +/+
Control
NPC 0.5 μM
INZ 0.5 μM
1:20 0.5 μM
H460
Control
NPC 0.5 μM
INZ 0.5 μM
1:20 0.5 μM

Figure S6

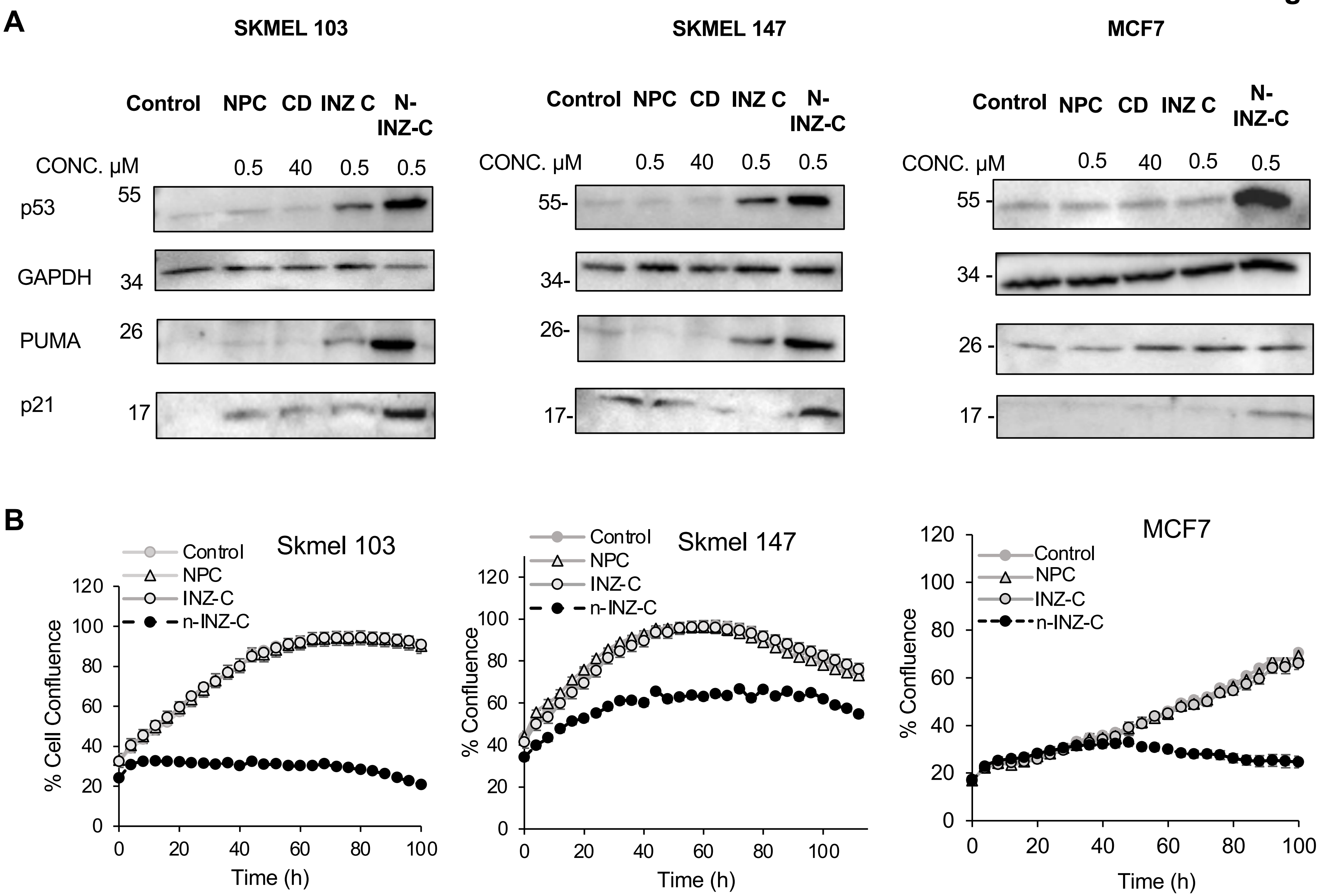
A
SKMEL 103
SKMEL 147
MCF7
Control
NPC
CD
INZ C
N-INZ-C
CONC. μM
0.5
40
0.5
0.5
p53
GAPDH
PUMA
p21
55
34
26
17
B
Skmel 103
Skmel 147
MCF7
Control
NPC
INZ-C
n-INZ-C
% Cell Confluence
% Confluence
Time (h)

Figure S7

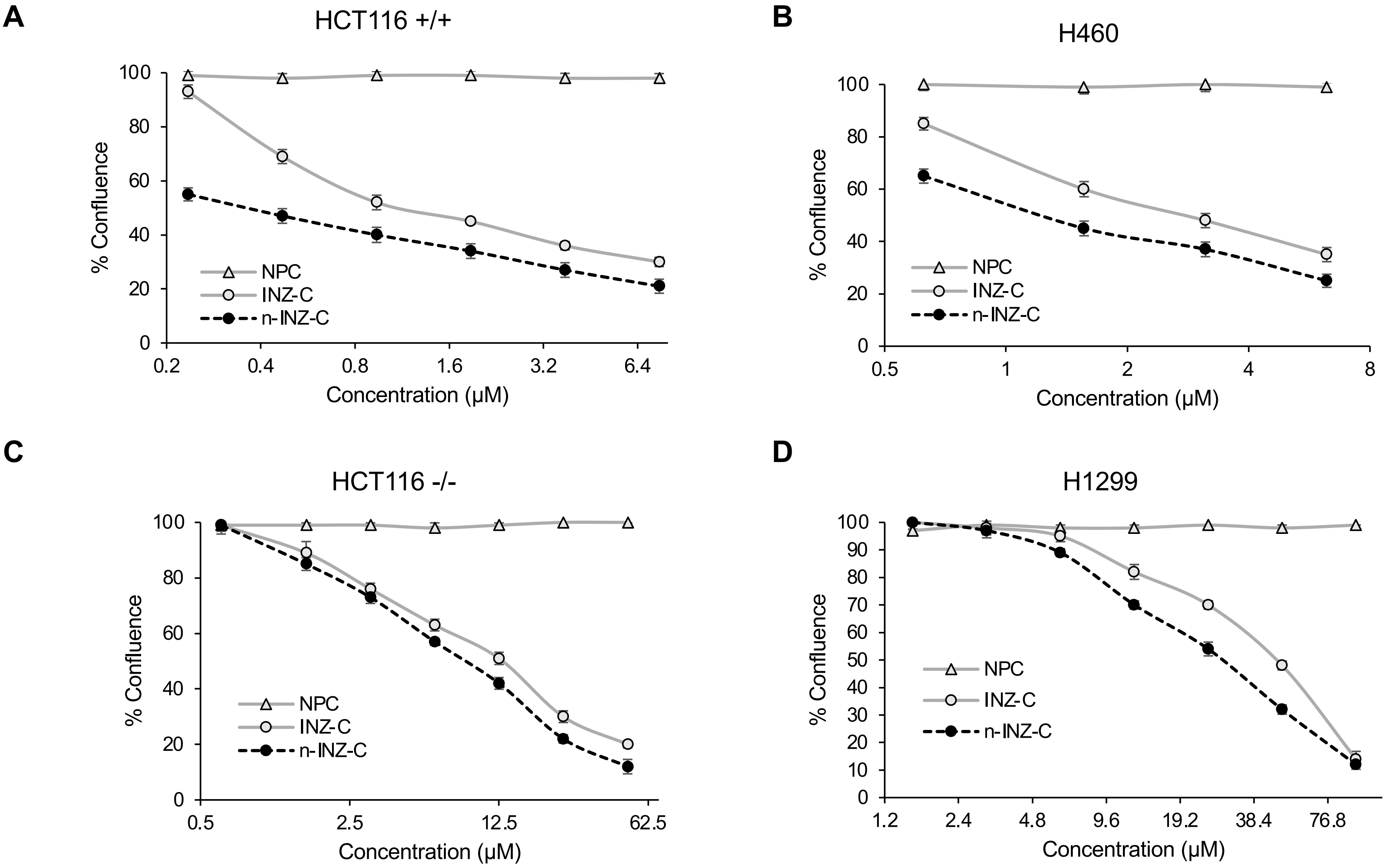
A
HCT116 +/+
% Confluence
Concentration (μM)
NPC
INZ-C
n-INZ-C
B
H460
% Confluence
Concentration (μM)
NPC
INZ-C
n-INZ-C
C
HCT116 -/-
% Confluence
Concentration (μM)
NPC
INZ-C
n-INZ-C
D
H1299
% Confluence
Concentration (μM)
NPC
INZ-C
n-INZ-C

Figure S8

A

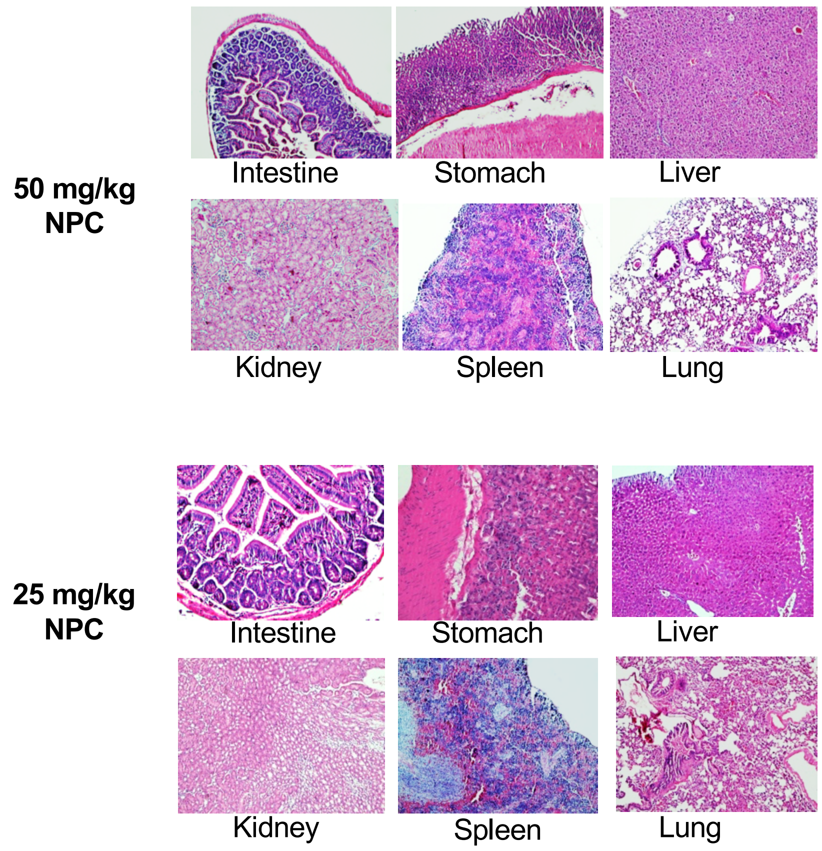
50 mg/kg
NPC
Intestine
Stomach
Liver
Kidney
Spleen
Lung
25 mg/kg
NPC
Intestine
Stomach
Liver
Kidney
Spleen
Lung

B

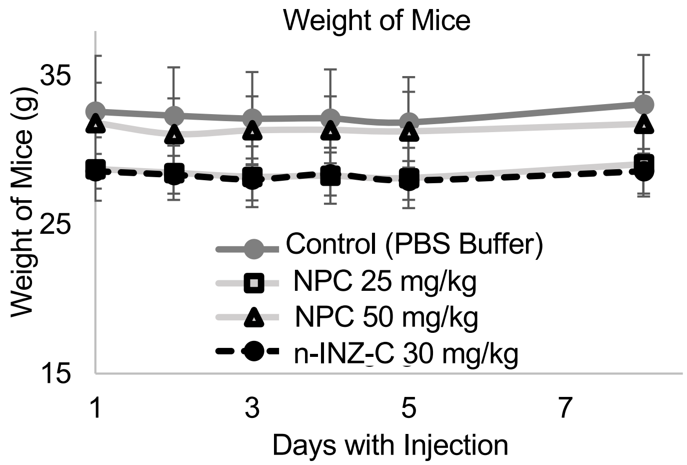
Weight of Mice
Weight of Mice (g)
35
25
15
1
3
5
7
Days with Injection
Control (PBS Buffer)
NPC 25 mg/kg
NPC 50 mg/kg
n-INZ-C 30 mg/kg

Figure S9

A

	**Peak Area**		
**nM**	ANA	INZ-C	Ratio
**0**	29422	8617.123	0.292
**18.25**	26418	8152.34	0.308
**37.5**	34657	14940	0.431
**75**	29808	16550	0.555
**150**	32918	20659	0.627
**300**	23265	22338	0.960
**1000**	30279	66637	2.200

B

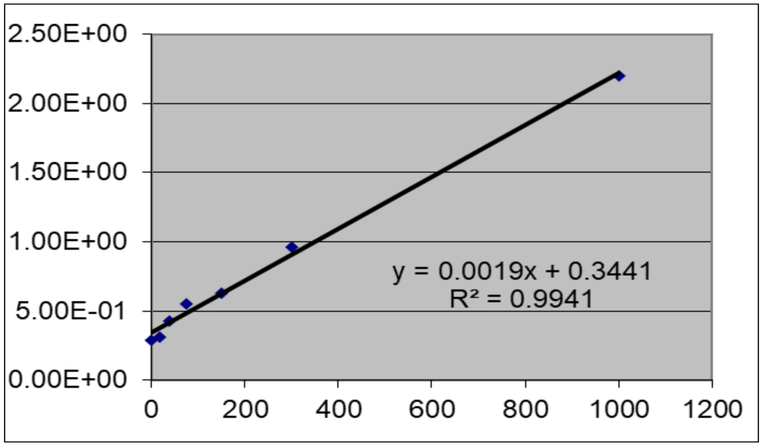

Figure S10

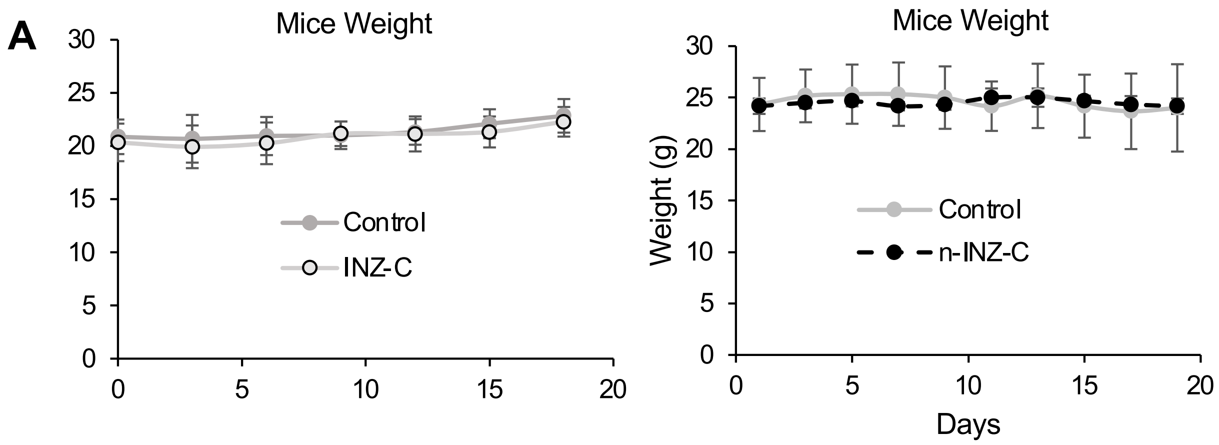

A
Mice Weight
30
25
20
15
10
5
0
0
5
10
15
20
Control
INZ-C
Mice Weight
Weight (g)
Control
n-INZ-C
Days

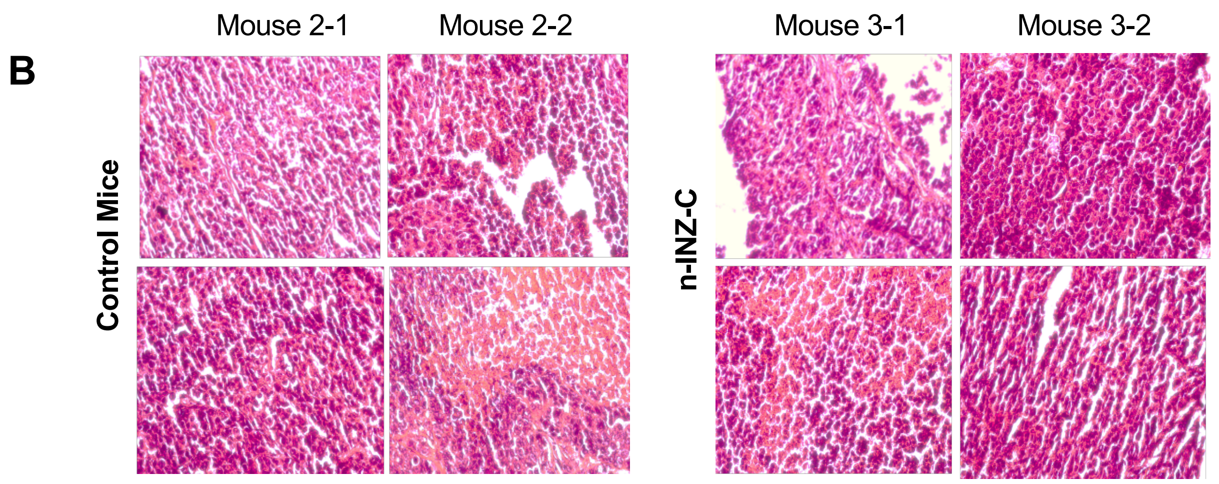

B
Mouse 2-1
Mouse 2-2
Mouse 3-1
Mouse 3-2
Control Mice
n-INZ-C
